# Porcine Respiratory and Reproductive Syndrome Virus Variants, Vietnam and China, 2007

**DOI:** 10.3201/eid1411.071676

**Published:** 2008-11

**Authors:** Youjun Feng, Tiezhu Zhao, Tung Nguyen, Ken Inui, Ying Ma, Thi Hoa Nguyen, Van Cam Nguyen, Di Liu, Quang Anh Bui, Long Thanh To, Chuanbin Wang, Kegong Tian, George F. Gao

**Affiliations:** Institute of Microbiology–Chinese Academy of Sciences, Beijing, People’s Republic of China (Y. Feng, Y. Ma, D. Liu, G.F. Gao); People’s Republic of China Animal Disease Control Center, Beijing (T. Zhao, C. Wang, K. Tian); National Center for Veterinary Diagnosis, Hanoi, Vietnam (T. Nguyen, T.H. Hguyen, V.C. Nguyen, L.T. To)

**Keywords:** Porcine respiratory and reproductive syndrome, PRRS virus, variant, Vietnam, China, dispatch

## Abstract

We characterized isolates from porcine respiratory and reproductive syndrome virus epidemics in Vietnam and China in 2007. These isolates showed ≈99% identity at the genomic level. Genetic analysis indicated that they share a discontinuous deletion of 30 aa in nonstructural protein 2, which indicates that identical variants emerged in Vietnam and China.

Porcine respiratory and reproductive syndrome (PRRS) is one of the most economically influential infectious diseases in the industry of swine cultivation. The etiologic agent is PRRS virus (PRRSV), a member of the family *Arteriviridae* in the order Nidovirales (*1**,**2*). Genomic analysis of PRRSVs has shown that the virus genome varies from 15 kb to 15.5 kb and comprises at least 8 open reading frames (ORFs) that encode nearly 20 mature proteins (*3*). PRRSVs with different geographic origins can be classified into 2 major genotypes, the European type (type I, EU-type) (*4*) and North American type (type II, NA-type) (*5*). Nonstructural protein 2 (Nsp2) (*6*) and glycoprotein 5 (encoded by ORF5) (*7*), are regarded as 2 regions of high heterogeneity that are involved in the pathogenicity of PRRSV strains.

In general, PRRSV has caused either respiratory failure in neonates or abortions in sows during sporadic PRRS outbreaks worldwide (*2*). The unprecedented large-scale PRRS outbreaks in 2006 swept over nearly half of the People’s Republic of China and involved >2,000,000 pigs, which posed great concern to the global swine industry and to public health (*8*). Subsequent genomic analysis showed that all the PRRSVs isolated from this outbreak (*8*) share a unique discontinuous deletion of 30 aa in Nsp2. In previous studies, only 2 virulent NA-type PRRSV strains (P129 and MN184) had been suggested to carry deletions in the Nsp2 protein (different from our 30-aa deletion), a highly variable region that contributes to the virulence and PRRSV genotyping (*9*,*10*).

A suspected PRRS outbreak was observed in Vietnam in 2007, and further spread of the disease has been found in China in 2007. To elucidate the characteristics of these outbreaks and the PRRSVs isolated, we conducted a detailed investigation.

## The Study

In March 2007, the suspected PRRS-like disease was initially found in Hai Duong Province, the northern province of Vietnam. The disease later spread to nearly the entire country and affected at least 65,000 pigs. Meanwhile, the recurrence of PRRSV infections in China was officially announced in May 2007 by the Chinese Ministry of Agriculture.

**Figure 1:**
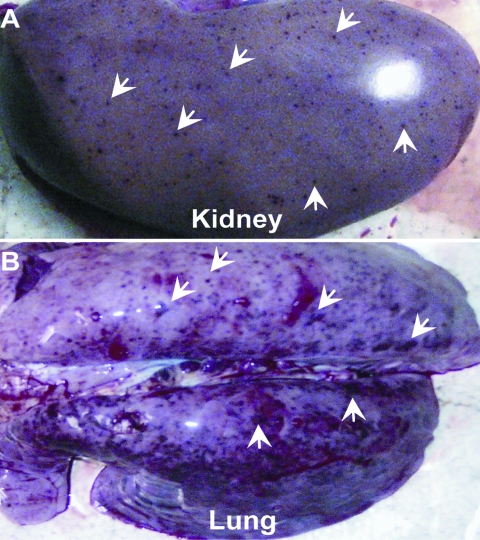
Pathologically dissected specimens from pigs infected by porcine respiratory and reproductive syndrome viruses. Blood spots in kidneys and lung hemorrhages are indicated by white arrows.

The affected pigs exhibit the clinical features of high fever, depression, and shivering. Viscera (e.g., brain, kidney, lung, heart, liver, and spleen) were sampled from those dead pigs from different provinces in Vietnam and China. Pathologic examination showed severe lesions viscera, for example, blood spots in the kidney and hemorrhages in the lung (Figure 1, panels A, B), findings similar to those observed in the 2006 PRRS outbreaks in China (*8*,*11*). The inocula (from each representative specimen) were propagated in Marc-145 cells (QIAGEN, Hilden, Germany) for the viral genomic RNA isolation. Based on our previous observation of the 2006 PRRS epidemics in China, we designed 2 pairs of specific primers (*Nsp2*-F: 5′-AAA GAC CAG ATG GAG GAG GA-3′ and *Nsp2*-R: 5′-GAG CTG AGT ATT TTG GGC GTG-3′; *orf5*-F: 5′-ATG TTG GGG AAG TGC TTG ACC-3′ and *orf5*-R: 5′-CTA GAG ACG ACC CCA TTG TTC CGC-3′). They correspond to the DNA fragment covering a putative discontinuous deletion of 30 aa in Nsp2 (666 bp), and ORF5 in full length, respectively. PCR-based detection showed that all the samples collected so far, are positive for PRRSV, but negative for African swine fever, classical swine fever, and foot-and-mouth disease viruses.

To test the virulence of Vietnam PRRSV isolates, 6 specific pathogen–free piglets were challenged with a representative strain (termed 07QN, which had been isolated from a dead pig in Quang Nam Province, Vietnam). The piglets were monitored for clinical signs every 6 hours. All procedures were conducted in a facility qualified with Biosafety Level 3 and approved by Vietnamese Committee for Approval of Drugs and Cosmetics ethics committee. As we expected, the piglets reproduced the serious symptoms similar to those observed in our field investigation, which indicated that the viral agent is highly pathogenic.

**Figure 2:**
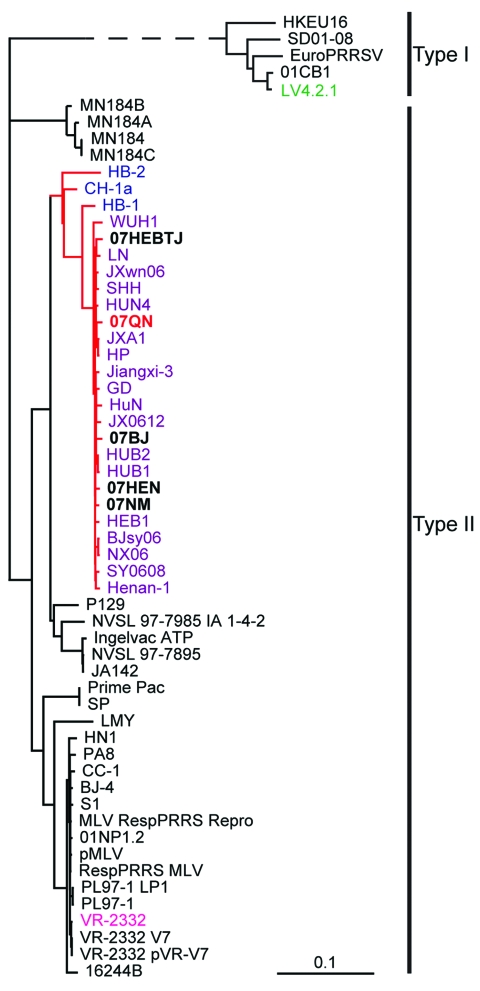
Whole genome–based phylogenetic relationship of porcine respiratory and reproductive syndrome viruses (PRRSVs) LV4.2.1, the prototype of type I (European PRRSV) (shown in green), and VR2332, the standard strain of type II (North American PRRSV) (shown in pink). Three Chinese isolates reported before 2006 (HB-1, HB-2, and CH-1a) are shown in blue. Chinese 2006 PRRSV isolates are shown in purple. Chinese 2007 isolates are shown in black **boldface**, and a representative of 2007 Vietnamese strains (07QN) is shown in red **boldface**. The 2007 Vietnamese and Chinese PRRSV isolates are classified into the same subclade of type II, as are all the 2006 Chinese PRRSV strains.

To better understand the genetic relationship of Vietnamese PRRSV isolates to the 2006 and 2007 Chinese isolates, 5 strains of PRRSVs (1 isolate from Vietnam, 07QN, and 4 newly isolated isolates from China, 07HEBTJ, 07BJ, 07HEN, and 07NM) were subjected to whole genome sequencing as described (*8*). The viral genomes were found to be >15,300 bp (GenBank accession nos. are available from the authors). Bioinformatics analysis further revealed that the 2007 Vietnamese isolate (07QN) and 2007 viruses (e.g., 07BJ) have 99% identity to 2006 PRRS isolates in China at the level of nucleic acid sequences. Whole genome–based phylogenetic relationship also showed that all these viral isolates are grouped into the same subclade in the type II genotype (Figure 2). All of the 2007 strains, together with those Chinese viruses collected in 2006, were found to be similar to 3 Chinese strains reported previously (HB-1, HB-2, and CH-1a) (*3*,*12*) and 1 NA-type virulent strain, P129 (*8,**9*).

In addition, >30 sequences of *orf5*, a highly variant gene, were sequenced, and ORF5-based genotyping also supported the classification of these 2007 isolates (from Vietnam and China) into a subgroup of type II, while far from other subclades with VR-2332, a prototype of type II (not shown). Moreover, the multiple alignments of Nsp2 demonstrated that the 2007 PRRSV isolates from Vietnam (51 total), together with 2007 Chinese strains (6 total) are nearly identical and share a discontinuous deletion of 30 aa at the position of 482 aa and 534–562 aa, which is consistent with deletions in the strains from the 2006 epidemic in China (*8*).

## Conclusions

PRRS has become a serious challenge to the global pig industry, causing serious economic losses (*2*). In particular, the unparalleled 2006 PRRS outbreaks engulfed China, and adult pigs were not exempt, which indicates that these variants may have evolved to exhibit new characteristics of pathogenicity (*8*). These outbreaks caused extensive concern worldwide (*13*). Even though the deletion of 30 aa in Nsp2 has been proposed as a potential virulence factor (*8*), the molecular mechanisms underlying its high virulence are yet to be elucidated.

Origin of these lethal variants in Vietnam is still obscure, although Kamakawa et al. (*14*) suggested that PRRSV may have been present in Vietnam before 1999. Convergent, but separate, evolution of PRRSVs in Vietnam and China may explain in part the emergence of the nearly identical PRRSV variants in these 2 neighboring countries, although this hypothesis does not rule out the possibility that 2006 Chinese PRRSV variants were transmitted into Vietnam and then circulated rapidly. Additionally, intraprovincial and interprovincial transportation of live pigs and the similar climate in Vietnam and China may have contributed to these outbreaks (*15*).

In summary, our findings provided robust evidence that nearly identical variants of NA-type PRRSVs are the causative pathogens that triggered PRRS epidemics in Vietnam and China in 2007. This finding highlights the importance of prevention and control of this highly transmissible infectious agent.
